# Critical appraisal of international guidelines for the prevention and treatment of pregnancy-associated venous thromboembolism: a systematic review

**DOI:** 10.1186/s12872-019-1183-3

**Published:** 2019-08-16

**Authors:** Jie Zheng, Qinchang Chen, Jing Fu, Yanling Lu, Tianjun Han, Ping He

**Affiliations:** 1 Department of Obstetrics, Guangzhou Women and Children’s Medical Center, Guangzhou Medical University, Guangzhou, China; 2 Department of Vascular Surgery, The First Affiliated Hospital of Sun Yat-sen University, Guangzhou, China; 3 Department of Gynecology and Obstetrics, Guangzhou Women and Children’s Medical Center, NO.402, Ren Min Middle Road, Yue Xiu District, Guangzhou, 510180 China

**Keywords:** Venous thromboembolism, Pregnancy, Prevention, Treatment

## Abstract

**Background:**

Pregnancy-associated Venous thromboembolism (VTE) is one of the most common causes of maternal morbidity and mortality in developed countries. In this study, we aimed to systematically review and critical appraisal of guidelines to compare the recommendations in pregnancy-associated VTE.

**Methods:**

Guidelines in English between January 1, 2009 and November 31, 2018 were searched using Medline via PubMed, as well as the guidelines’ website. The guidelines containing the recommendations on pregnancy-associated VTE were included. Through the Appraisal of Guidelines Research and Evaluation II (AGREE II) instrument, three reviewers appraised the quality of the included guidelines. The recommendations were also summarized and compared to analyze the consistency.

**Results:**

Fifteen guidelines from 13 organizations were included. Ten guidelines from nine organizations, namely, ACCP, ANZJOG, ASH, Australia, ESC, Korea, RCOG, SASTH, SOCC, were regarded as “strongly recommended for use in practice”. Most of the included guidelines scored low in lower scores in domain 3 (Rigor of development) and domain 6 (Editorial independence). Recommendations on prevention are contained in ten guidelines while treatment are included in seven. The main conflicting recommendations were mainly at the anticoagulant choice for prevention on pregnant women and prevention after cesarean section. The duration of VTE treatment in pregnant women was also controversial.

**Conclusions:**

In summary, the quality of pregnancy-associated VTE guidelines varied widely, especially in Rigor of development and Editorial independence. Recommendations were inconsistent both in prevention and treatment across guidelines. Increased efforts are required to provide high-quality evidence specific to the pregnancy population. Guideline developers should also pay more attention to methodological quality.

**Electronic supplementary material:**

The online version of this article (10.1186/s12872-019-1183-3) contains supplementary material, which is available to authorized users.

## Background

Pregnancy-associated Venous thromboembolism (VTE), including deep vein thrombosis (DVT) and pulmonary embolism (PE), is one of the most common causes of maternal morbidity and mortality in developed countries [1]. As a pro-inflammatory condition with activation of endothelial cells, pregnancy poses a higher risk of VTE [2]. When compared with the nonpregnant women, the risk is increased up to ten-fold in pregnancy [3, 4]. During the postpartum period, especially after cesarean section, the daily risk of VTE is nearly thirty-fold compared to nonpregnant women [3, 5]. However, clinical decisions about the management of pregnancy-associated patients are challenging and complex. When clinical management is applied, further considerations are needed regarding the potential complications of fetus and pregnant women, such as pregnancy loss, congenital malformations, and major maternal hemorrhage [6].

There are many clinical practice guidelines (CPGs) published for pregnancy-associated VTE patients. Using the method of evidence base, these guidelines attempted to summarize and organize the existing evidence to provide recommendations on clinical decisions. Due to the paucity of related studies of high quality, CPGs are mainly based on observation studies rather than randomized controlled trials (RCTs). Moreover, some studies are not specifically targeted at the pregnancy population, just the extrapolation from results in nonpregnant patients. The lack of RCTs can be explained by the difficulty of conducting RCTs with adequate statistical power due to the low rate of thrombosis among women identified as having a high risk of VTE. The guidelines at high quality are commonly believed to optimize clinical practice and improve patient outcomes [7, 8]; nevertheless, the adoption hinges on how they are developed. To the best of our knowledge, evaluation of the quality of CPGs for pregnancy-associated VTE has not been previously undertaken.

Therefore, we aimed to systematically assess the quality of pregnancy-associated VTE guidelines using the Appraisal of Guidelines for Research & Evaluation II (AGREE II) instrument and evaluate the consistency of recommendations. [9]

## Methods

A systematic review was undertaken using the Cochrane methodology [10].

### Search strategies

A systematic search was undertaken to search the guidelines related to pregnancy-associated VTE. Briefly, relevant guidelines were obtained by searching MEDLINE and EMBASE. In addition, four guideline-related databases, the Guidelines International Network (G-I-N) International Guideline Library, the National Guidelines Clearinghouse (United States), the Canadian Medical Association Infobase (Canada) and the National Library for Health (United Kingdom), were searched for any guidelines, which might have been missed by systematic searches. We limited the search time from January 1, 2009 to November 31, 2018. Details on the search terms and syntax are provided in Additional file 1: Table S1.

### Selection criteria

The Institute of Medicine defines CPGs as “systematically developed statements to assist practitioner and patient decisions about appropriate health care for specific clinical circumstances.” [11]. According to the Institute of Medicine, articles were considered if they met the definition. In addition, we chose guidelines using the following inclusion criteria: (1) the guidelines contain recommendations on the management of pregnancy-associated VTE; (2) the guidelines are published in English; and (3) the full text can be available online. If doubt existed whether guidelines met the criteria or not, discussions would be held to reach consensus agreements.

The guidelines were excluded for the following reasons: (1) historical versions of guidelines had been subsequently updated; (2) the topic is only mentioned in the guidelines; (3) unpublished guidelines, conference paper, discussion paper, draft and opinions are excluded.

### Quality appraisal of the guidelines

We assessed the quality of each included guideline using the AGREE II instrument [9]. AGREE II is an international validated tool to appraise guideline development, consisting of 23 items organized into 6 domains: scope and purpose, stakeholder involvement, rigor of development, clarity of presentation, applicability and editorial independence (Details in Additional file 2: Table S2). Two reviewers (JZ and QCC) independently rated each item on a seven-point Likert scale from 1 (strongly disagree) to 7 (strongly agree). When it is poorly reported or without any information relevant to the item, a score of 1 is given, and when the item meets all the criteria, a score of 7 is given. If the two reviewers rated items with a difference of more than 2 points, a third reviewer (PH) was asked to decide the final score. After summing all the scores of each item in a domain, the final rigor score for each domain was converted to a percentage by calculating in this formula:

$$ \frac{\mathrm{Obtained}\ \mathrm{score}-\mathrm{Minimum}\ \mathrm{possible}\ \mathrm{score}}{\mathrm{Maximum}\ \mathrm{possible}\ \mathrm{score}-\mathrm{Minimum}\ \mathrm{possible}\ \mathrm{score}}\ast 100\% $$

Thresholds were determined to assess guideline overall quality. We considered a guideline as “strongly recommended for use” if majority of domains of it scored over 60%, as “recommended with modifications” if the majority of domains scored between 30 and 60%, as “not recommended for use” if the majority of domains were below 30%.

Data collection and recommendations synthesis.

One reviewer (JZ) extracted the information about guideline characteristics, including year of publication, country/region, development team, target population, target users, and funding organization (Additional file 3: Table S3). The recommendations on the management of pregnancy-associated VTE were extracted by another reviewer (QCC). We compared the recommendations to identify similarities and discrepancies, and the information was tabulated.

## Results

### Search results

**Fig. 1:**
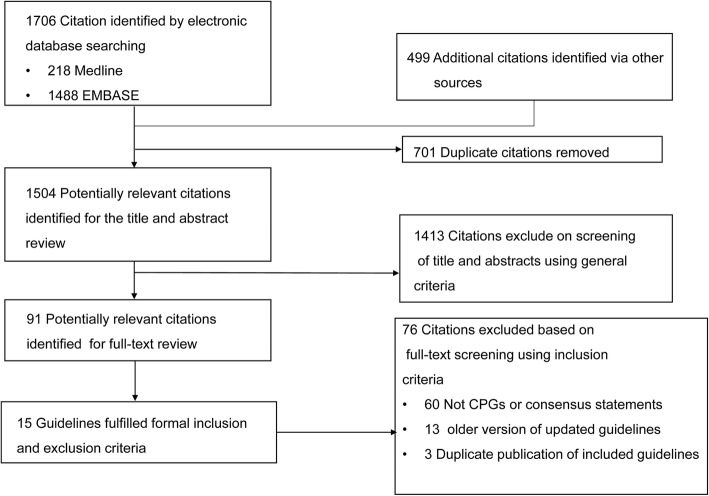
Flow diagram of the identification process for guidelines on prevention and treatment in pregnancy-associated VTE

One thousand five hundred and four citations were retrieved, of which 1413 citations were excluded after screening the titles and abstracts. The remaining 91 citations were assessed for full-text articles, and many of them were excluded after applying the inclusion and exclusion criteria (Fig. 1). Finally, 15 guidelines from 13 organizations (American College of Chest Physicians (ACCP) [12], American College of Obstetricians and Gynecologists (ACOG) [13], Australian and New Zealand Journal of Obstetrics and Gynaecology (ANZJOG) [14, 15], American Society of Hematology (ASH) [16]; Australia [17], Asian Venous Thrombosis Forum (AVTF) [18], European Society of Cardiology (ESC) [19], Working Group in Women’s Health of the Society of Thrombosis and Haemostasis (GTH) [20], Journal of Obstetric, Gynecologic & Neonatal Nursing (JOGNN) [21], Korea [22], Royal College of Obstetricians and Gynaecologists (RCOG) [23, 24], Southern African Society of Thrombosis and Haemostasis (SASTH) [25], Society of Obstetricians and Gynaecologists of Canada (SOGC) [26]) were included in this study.

### Characteristics of the guidelines

The characteristics of the included guidelines are shown in Table 1. These guidelines were published between 2011 and 2018, among which four guidelines were regional, two were published by Australia and New Zealand [14, 15], one was from Asia [18] and one was from Europe [19]. Four guidelines were from the USA [12, 13, 16, 21]; the remaining guidelines were from Australia [17], Germany [20], Korea [22], Unite Kingdom [23, 24], South Africa [25], Canada [26], respectively. Eleven guidelines [12–14, 16–19, 21–23, 25] contained recommendations for the prevention of pregnancy-associated venous thromboembolism, while seven guidelines [12, 15, 16, 19, 20, 24, 26] included treatment. Five guidelines grated the strength of the recommendations by using the Grading of Recommendations Assessment, Development and Evaluation (GRADE) approach [12, 14–17, 19, 26]. The information about conflicts of interest (COI) was only reported in six guidelines [12, 16–18, 22–24].

**Table 1:** Included clinical practice guidelines on pregnancy-associated venous thromboembolism

CPGs	Year	Country/ Region	Evidence base	Topics covered	No. of reference	Guideline Page	Strength of the recommendations	Status	Conflicts of interest
ACCP [12]	2012	USA	Yes	Treatment Prevention	343	46	GRADE	Updated	EI; SCI
ACOG [13]	2013	USA	Not reported	Prevention	69	12	Not reported	Updated	Not reported
ANZJOG [14, 15]	2011	Australia New Zealand	Yes	Prevention Treatment	136	20	GRADE	New	Not reported
ASH [16]	2018	USA	Yes	Prevention Treatment	243	43	GRADE	New	SCI, EI
Australia [17]	2012	Australia	Yes	Prevention	51	11	GRADE	New	SCI
AVTF [18]	2016	Asia	Not reported	Prevention	143	20	Not reported	Updated	Not reported
ESC [19]	2011	Europe	Yes	Treatment Prevention	254	51	GRADE	Updated	SCI, EI
GTH [20]	2016	Germany	Not reported	Treatment	16	125	Not reported	New	Not reported
JOGNN [21]	2016	USA	Not reported	Prevention	12	34	No	New	Not reported
Korea [22]	2014	Korea	Not reported	Prevention	8	36	Not reported	Updated	SCI
RCOG [23, 24]	2015	UK	Yes	Prevention Treatment	355	72	Standard grading scheme	Updated	SCI,EI
SASTH [25]	2013	South Africa	Not reported	Prevention	7	22	No	New	Not reported
SOGC [26]	2014	Canada	Yes	Diagnosis Treatment	27	187	GRADE	New	Not reported

*ACCP* American College of Chest Physicians, *ACOG* American College of Obstetricians and Gynecologists, *ANZJOG* Australian and New Zealand Journal of Obstetrics and Gynaecology, *ASH* American Society of Hematology, *AVTF*, Asian Venous Thrombosis Forum; *EI* editorial independence declared, *ESC* European Society of Cardiology, *GTH* Working Group in Women’s Health of the Society of Thrombosis and Haemostasis, *JOGNN* Journal of Obstetric, Gynecologic & Neonatal Nursing, *RCOG* Royal College of Obstetricians and Gynaecologists, *SASTH* Southern African Society of Thrombosis and Haemostasis, *SCI* statement about conflicts, *SOGC* Society of Obstetricians and Gynaecologists of Canada

### Guideline appraisal

**Fig. 2:**
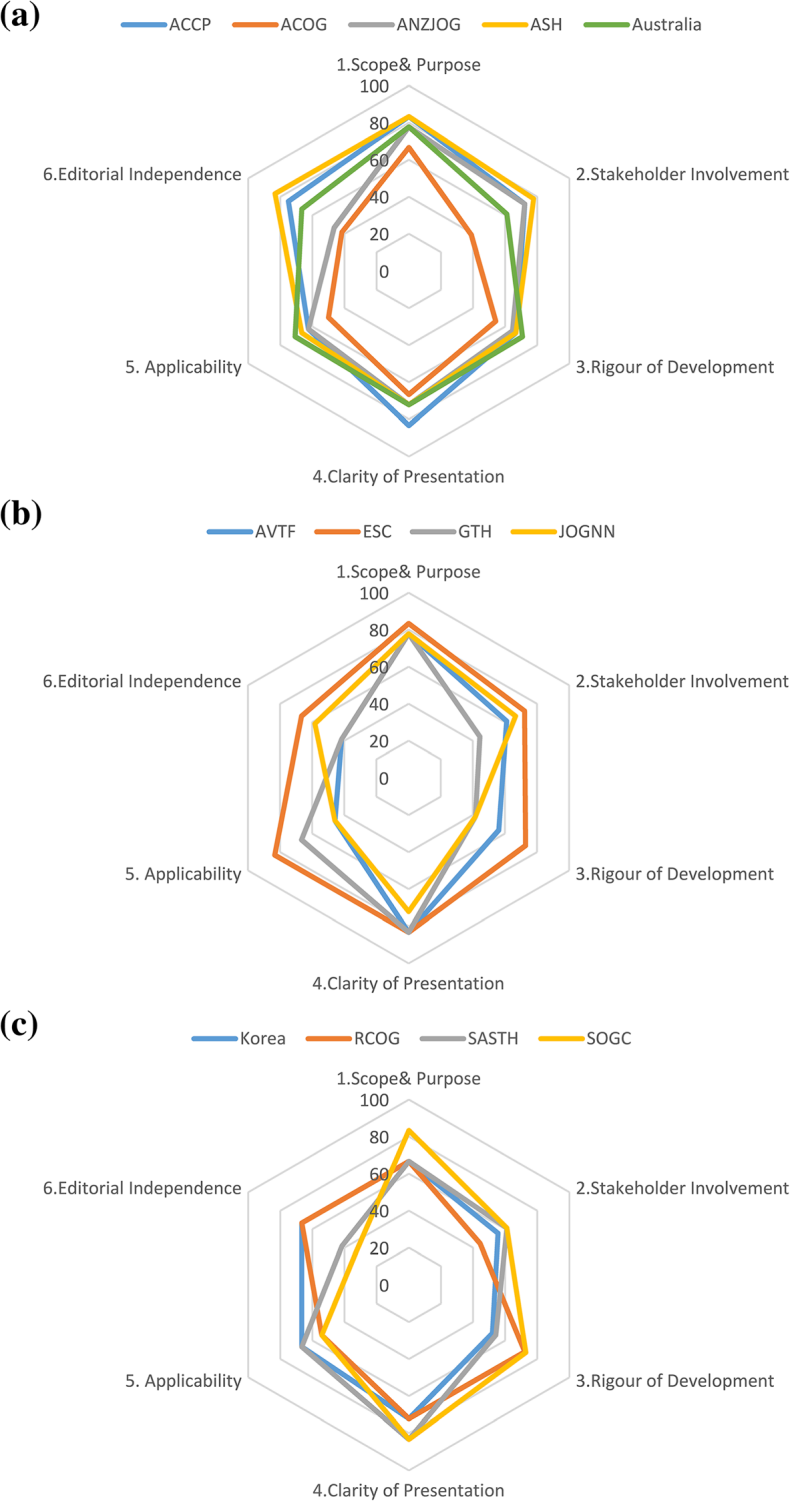
Final Domain Scores. AGREE II scores are plotted for each guideline for comparison. The higher percentage meant the better quality in the domain and was mapped towards the outer perimeter (closer to 100%)

Figure 2 shows the final scores of six domains in the included guidelines. To present the results of the guideline appraisal, a radar chart was selected. When the percentage is higher, the graph of the guidelines mapped toward outer and meant the better quality. As shown, ACCP, ANZJOG, ASH, Australia, ESC and RCOG had relatively higher scores in most domains [12, 14–17, 19, 23, 24]. Most guidelines scored higher in domain 1 (Scope & purpose) and domain 4 (Clarity of presentation); nevertheless, some of the guidelines had lower scores in domain 3 (Rigor of development) and domain 6 (Editorial independence). Only eight guidelines reported the review protocol [12, 14–17, 19, 23, 24, 26], and the information about COI was mentioned in six guidelines [12, 16, 17, 19, 22–24]. Eight guidelines from seven organizations, namely, ACCP, ANZJOG, ASH, Australia, ESC, Korea, RCOG, SASTH, SOCC, were regarded as “strongly recommended for use in practice” [12, 14–17, 19, 22–26]. Four remaining guidelines were scored as “recommended for use with some modification” while no guideline was regarded as “not recommended for use in practice”. The raw data of guideline appraisal was shown in Additional file 4: Table S4.

### Recommendations on approaches to prevention

Eleven guidelines contained recommendations on prevention for pregnancy-associated VTE patients [12–14, 16–19, 21–23, 25]. The important recommendations were collected in Table 2. For the anticoagulant choice for pregnant patients, Low Molecular Weight Heparin (LMWH) was the main choice and recommended by all the guidelines. Conflicting recommendations were observed for the other anticoagulants. The ACCP guidelines [12] recommended LMWH rather than Unfraction Heparin (UFH) for prevention, while UFH was recommended in the JOGNN guidelines [21]. The Korean guidelines [22] recommended against Warfarin in the pregnancy population while they were supported in the Australia guidelines [17]. For the VTE at low risk, all the guidelines recommended against the prevention. For moderate to high risk, prophylactic dose LMWH was recommended as the main choice, and two guidelines [18, 19] recommended mechanical prophylaxis. Warfarin was only advocated in Australia guidelines [17]. After cesarean section (CS), LWMH was recommended by five guidelines [12, 17, 19, 22, 23], while Warfarin was recommended by Australia guidelines [17] and Korea guidelines [22]. For CS patients with VTE at low risk, three guidelines [12, 22, 24] recommended against the prevention while Australia guidelines [17] supported. For moderate risks, prophylactic LMWH for 7 days seemed to be the main choice, while mechanical prophylaxis was also recommended by three guidelines [12, 14, 17]. For high risk, the duration of prophylaxis was 6 months. Apart from LMWH, adjusted therapeutic dose warfarin was recommended by two guidelines [14, 17]. Five guidelines [12–14, 19, 22] recommended screening for inherited thrombophilia (IT). The prevention of IT was recommended against three guidelines [12–14], only supported in Korea guidelines [22].

**Table 2:** Summary of recommendations on prevention of obstetric-associated venous thromboembolism

	ACCP [12]	ACOG [13]	ANZJOG [14]	ASH [16]	Australia [17]	AVTF [18]	ESC [19]	JOGNN [21]	Korea [22]	RCOG [23]	SASTH [25]
Pregnant patients											
Anticoagulant choice	LMWH UFH (×)		LMWH		LMWH Warfarin	LMWH	LMWH	LMWH UFH	LMWH Warfarin(×)	LMWH	LMWH NOAC(×)
Low risks	×		×	×	×	×	×	×			
Moderate to high risk	Prophylactic- or intermediate dose LMWH		Prophylactic LMWH	Prophylactic LMWH	LMWH or Adjusted dose warfarin	LMWH or mechanical prophylaxis	LMWH or mechanical prophylaxis	Prophylactic-dose LMWH or UFH			
Cesarean section											
Anticoagulant choice	LMWH				LMWH warfarin		LMWH		warfarin LMWH	LMWH	
Low risks	×				√				×		×
Moderate risks	prophylactic LMWH or mechanical prophylaxis		mobilize early, mechanical prophylaxis		LMWH for 5–7d or mechanical prophylaxis		LMWH for 7d	prophylactic-dose LMWH or UFH 6w		LMWH for 10d	LMWH
High risk	prophylactic LMWH and mechanical prophylaxis for 6w		prophylactic LMWH or Warfarin for 6w		LMWH or adjusted therapeutic dose warfarin to 6w		LMWH for 6w and mechanical prophylaxis	treatment-dose LMWH or UFH 6w		LMWH for 6w	
Inherited thrombophilia											
Screening	×	×	×				×		×		
Prevention	×	×	×						√	√	

ACCP, American College of Chest Physicians; ACOG, American College of Obstetricians and Gynecologists; ANZJOG, Australian and New Zealand Journal of Obstetrics and Gynaecology; AVTF, Asian Venous Thrombosis Forum; ESC, European Society of Cardiology; JOGNN, Journal of Obstetric, Gynecologic & Neonatal Nursing; LMWH, Low Molecular Weight Heparin; NOAC, Novel Oral Anticoagulants; SASTH, Southern African Society of Thrombosis and Haemostasis; UFH, Unfraction Heparin

### Recommendations on approaches to treatment

Seven organizations provided recommendations on treatment [12, 15, 16, 19, 20, 24, 26]. The recommendations were collected in Table 3. LMWH and UFH were mainly recommended. ESC guidelines [19] recommended LMWH for low risk and UFH for high risk, while GTH guidelines [20] preferred LMWH. Novel oral anticoagulants (NOACs) and vitamin K antagonist (VKA), such as Warfarin, were not recommended. The duration of treatment was recommended for 3 months by three guidelines [12, 20, 25] while that was 6–8 months in the ANZJOG guidelines [15] and 6 w-3 m in the RCOG guidelines [23]. The ANZJOG guidelines [15] recommended compression stocking for 2 years, and the SOCG guidelines [26] also supported mechanical prophylaxis. Ven cava filters were recommended in patients with iliac vein VTE, with proven DVT and recurrent PE in RCOG guidelines [24], while they were recommended in patients with contraindications for anticoagulation [15, 26]. For delivery patients, the recommendation was rare and only mentioned in ACCP guidelines [12]. For lactating women, LMWH and VKA were the main anticoagulant choices. The duration was recommended for 6 weeks.

**Table 3:** Summary of recommendations on treatment of obstetric-associated venous thromboembolism

	ACCP [12]	ANZJOG [15]	ASH [16]	ESC [19]	GTH [20]	RCOG [24]	SOGC [26]
Pregnant patients							
Anticoagulant choice	LMWH, UFH NOACs(×)	LMWH, UFH, VKA (×)	LMWH (prefer), UFH	UFH (high risk) LMWH (low risk)	LMWH (prefer), UFH VKA (×), NOACs (×)	LWMH	LMWH, VKA (×, unless special situation), NOACs (×)
Duration	3 m	6-8 m			3 m	6w-3 m	3 m
Mechanical prophylaxis		Compression stocking 2y					√
Vena cava filters		Acute DVT with contra-indications for anticoagulation				Patients with iliac vein VTE, with proven DVT and recurrent PE	Acute DVT with contra-indications for anticoagulation
Thrombolysis		Only life-threatening DVT	Not recommend		Only life-threatening PE	Massive PE	Only life-threatening DVT
Delivery patients	Discontinuation of LMWH at least 24 h					Intravenous UFH for 24 h	
Lactating women							
Anticoagulant choice	VKA, UFH, LMWH				Warfarin, LMWH	LMWH, Warfarin (X)	
Duration	6w	6w			6w	6w-3 m	6w

*ACCP* American College of Chest Physicians, *ANZJOG* Australian and New Zealand Journal of Obstetrics and Gynaecology, *ASH* American Society of Hematology, *DVT* Deep Vein Thrombosis, *ESC* European Society of Cardiology, *GTH* Working Group in Women’s Health of the Society of Thrombosis and Haemostasis, *LMWH* Low Molecular Weight Heparin, *NOAC* Novel Oral Anticoagulants, *PE* Pulmonary Embolism, *RCOG* Royal College of Obstetricians and Gynaecologists, *SOGC* Society of Obstetricians and Gynaecologists of Canada, *UFH* Unfraction Heparin, *VKA* Vitamin K antagonist

## Discussion

To the best of our knowledge, this is the first guideline appraisal to systematically synthesize and appraise pregnancy-associated VTE. Finally, 15 guidelines from 13 organizations reporting the recommendations related to prevention or treatment of pregnancy-associated VTE were included. The scores assessed by AGREE II varied both between guidelines across domains and between different domains in one guideline. Domain 1 (Scope & purpose) and domain 4 (Clarity of presentation) obtained relatively high scores, while the scores in domain 3 (Rigor of development) and domain 6 (Editorial independence) were low. The information about the evidence base was only mentioned in six guidelines [12, 14–17, 19, 23–25]. Most guidelines did not report the strength of the recommendation and the quality of evidence. There was too little information about the funding body and COI among guideline development members. The inconsistent recommendations across pregnancy-associated VTE were observed both in the prevention and treatment. For prophylaxis in pregnant patients, the Australia guidelines [17] suggested Warfarin to be an anticoagulant choice, while this was recommended against Korean guidelines [22]. After cesarean section, only Australia guidelines [17] recommended for prevention at low risk. Conflicting recommendations were also observed in the duration of treatment.

The conflicting recommendations might result from the process of guideline development. CPGs are developed to assist the clinician decision under different clinical settings. The proper use of CPGs at high quality is essential to reduce practice variation and improve patient outcome [11]. Although many guidelines have been published in recent years, the impact of CPGs on one clinical decision was limited. In contrast, more and more concern occurred toward the quality of the guidelines and consistency in recommendations. To date, a great number of guidelines have been published on pregnancy-associated VTE, while no appraisal of the guidelines has been published. After the assessment by the AGREE II instrument, the quality of guidelines varied widely both in different domains between guidelines. ACCP, ANZJOG, ASH, Australia, ESC and RCOG scored high in most domains, while there were four guidelines scored as “recommended for use with some modification”. Moreover, the score differed in domain 3 (Rigor of development) and domain 6 (Editorial independence) because of the difference in the method for systematic review and COI statement. It is worth noting that transparency among guidelines developers impacts recommendation formation. In a study of opioid treatment for chronic pain, the organizations seemed to oppose the guidelines on opioids when they were funded by opioid companies [27]. In the process of guideline development, high methodological quality is of great importance, while insufficient attention has been paid.

Although pregnancy-associated VTE is uncommon, it remains a leading cause of maternal morbidity and mortality worldwide [1, 2, 28]. Due to potential complications both in the fetus and maternal, the management of pregnancy-associated VTE is difficult. In this study, conflicting recommendations were observed both in prevention and treatment. LMWH is regarded as the main anticoagulant choice for the prevention of pregnancy in women. Warfarin is the major point in dispute. Australia guidelines [17] recommended adjusted dose warfarin in pregnancy prophylaxis while recommended against Korean guidelines [22]. Australia guidelines did not specially provide specific evidence for the recommendations [17]. In contrast, the Korean guidelines [22] provided the recommendation explicitly that warfarin is contraindicated during pregnancy as well as the reference [29]. Thromboprophylaxis might benefit women at risk for VTE after caesarean [30, 31]. Four guidelines contained recommendations on CS patients at low risk, of which the Australia guidelines [17] recommended prevention; the remaining three guidelines [12, 22, 24] recommended prevention. This guideline [17] was not specifically provided to the pregnancy population. Moreover, the guideline development methodology was ADAPTED, rather than the GRADE method, which might result in conflicting recommendations [32, 33]. Meanwhile, the challenge in pregnancy-associated VTE has led to the paucity of high-quality research. Though many guidelines published the recommendations using the method of evidence base, the quality of evidence was relatively low. Most of the recommendations were based on larger observational research or were just extrapolated from studies in a nonpregnancy population. The lack of research in pregnant women, especially studies with high quality, has resulted in inconsistencies in recommendations.

Without the clear-cut evidence, the consistency of recommendations will be more sensitive to the methodological method and conflicts of interest.

The strength of this study is a comprehensive literature search. We carefully collected the information about the guideline development process and consideration about the quality by judging each item in the AGREE-II instrument, which is hopeful for enhancing the quality of guidelines. It is of great importance to perform the guideline appraisal, especially for the countries without their own guidelines on managing VTE in pregnancy. Guideline appraisal is essential to determine the guidelines with high quality and the recommendations with agreement from most guidelines, which are useful on the extent to the countries without their own guidelines. However, our study has some potential limitations. First, only guidelines published in English were reviewed, and we might overlook the other guidelines written by other languages. Second, the appraisal of CPGs was merely based on the information reported by the authors. Hence, some items in AGREE II could have a low score because of the lack of related information, even though the authors had the complete process during guideline development. Moreover, most guidelines included did not state the funding sources. It was difficult to evaluate whether there was an influence from the commercial industry. Third, AGREE II is a tool used to access the quality of the guideline development instead of the quality of the evidence. Recommendations from high-score CPGs might be based on weak evidence and vice versa.

Fourth, because the number of guidelines on pregnancy VTE is limited, the guideline that is not specifically targeted on the pregnancy population but still with related recommendations was also included in this study [17]. During the guideline appraisal, each item would presumably be assessed for the whole group of patients, which might impact the assessment of guideline quality and make a difference in reliability when compared with the guidance for pregnant women specifically.

## Conclusions

In summary, the quality of pregnancy-associated VTE guidelines varied widely, especially in Rigor of development and Editorial independence. Recommendations were inconsistent both in prevention and treatment across guidelines. Increased efforts are required to provide high-quality evidence specific to the pregnancy population. Guideline developers should also pay more attention to methodological quality.

### Guidelines included

ACCP [12]

ACOG [13]

ANZJOG [14, 15]

ASH [16]

Australia [17]

AVTF [18]

ESC [19]

GTH [2O]

JOGNN [21]

Korea [22]

RCOG [23, 24]

SASTH [25]

SOGC [26]

## Additional files

Additional file 1: **Table S1.** Search strategies. (DOCX 15 kb)

Additional file 2: **Tables S2.** Structure and content of the AGREE instrument. (DOCX 15 kb)

Additional file 3: **Tables S3.** Data extraction template. (DOCX 14 kb)

Additional file 4: **Tables S4.** Raw Data. (DOCX 16 kb)

## Data Availability

The dataset supporting the conclusions of this article is included within the article.

## Abbreviations

ACCP: American college of chest physicians
ACOG: American College of Obstetricians and Gynecologists
AGREE: Appraisal of guidelines research and evaluation
ANZJOG: Australian and New Zealand journal of obstetrics and Gynaecology
ASH: American Society of Hematology
AVTF: Asian venous thrombosis forum EI Editorial independence declared
ESC: European society of cardiology
GTH: Working Group in Women’s health of the Society of Thrombosis and Haemostasis
JOGNN: Journal of obstetric, Gynecologic & Neonatal Nursing
RCOG: Royal College of Obstetricians and Gynaecologists
SASTH: Southern African Society of Thrombosis and Haemostasis
SCI: Statement about conflicts
SOGC: Society of Obstetricians and Gynaecologists of Canada
