# Supplementary material for: Critical appraisal of international guidelines for the prevention and treatment of pregnancy-associated venous thromboembolism: a systematic review
Source: BMC Cardiovasc Disord. 2019 Aug 16;19:199. doi: 10.1186/s12872-019-1183-3 (PMC6698012; doi:10.1186/s12872-019-1183-3)
Supplement: Supplementary file 1 — Table S1. Search strategies. (DOCX 15 kb) [file 12872_2019_1183_MOESM1_ESM.docx]

**Additional file 1: Table S1. Search Strategies**

Search strategies Medline

1. "guideline"[Publication Type]
2. "guidelines as topic"[MeSH Terms]
3. "guideline"[All Fields]
4. 1 OR 2 OR 3
5. "Venous Thromboembolism"[Mesh]
6. Thromboembolism, Venous
7. 5 OR 6
8. "Pregnancy"[Mesh]
9. Pregnancies
10. Gestation
11. 8 OR 9 OR 10
12. 4 AND 7 AND 11

Search strategies EMBASE

1. Thromboembolism/or Vein Thrombosis/
2. (((venous or vein) adj (thrombosis or thrombus or thromboembolism)) or (dvt or vte) or ((pulmonary or lung) adj (embolism or emboli))).ti,ab.
3. or/1-2
4. exp Pregnancy/
5. exp Pregnant Women/
6. pregnan*.mp.
7. gestation*.mp.
8. or/4-7
9. exp practice guideline/
10. guideline$.tw.
11. consensus.tw.
12. position statement$.tw.
13. exp health care policy/ or exp policy/
14. recommendation$.tw.
15. or/9-14
16. 3 and 8 and 15
