# Supplementary material for: Critical appraisal of international guidelines for the prevention and treatment of pregnancy-associated venous thromboembolism: a systematic review
Source: BMC Cardiovasc Disord. 2019 Aug 16;19:199. doi: 10.1186/s12872-019-1183-3 (PMC6698012; doi:10.1186/s12872-019-1183-3)
Supplement: Supplementary file 4 — Tables S4. Raw Data. (DOCX 16 kb) [file 12872_2019_1183_MOESM4_ESM.docx]

Table S5. AGREE II scores on each domain for pregnancy-associated venous thromboembolism

| **CPGs** | **Scope and**  **purpose (%)** | **Stakeholder**  **involvement (%)** | **Rigor of**  **development (%)** | **Clarity of**  **presentation (%)** | **Applicability (%)** | **Editorial**  **independence (%)** |
| --- | --- | --- | --- | --- | --- | --- |
| **ACCP**^12^ | 83.33 | 72.22 | 66.67 | 83.33 | 62.5 | 75 |
| **ACOG**^13^ | 66.67 | 38.89 | 54.17 | 66.67 | 50 | 41.67 |
| **ANZJOG** ^14,15^ | 77.78 | 72.22 | 64.58 | 72.22 | 62.5 | 46.47 |
| **ASH**^16^ | 83.33 | 77.78 | 66.67 | 72.22 | 66.67 | 83.33 |
| **Australia**^17^ | 77.78 | 61.11 | 70.83 | 72.22 | 70.83 | 66.67 |
| **AVTF**^18^ | 77.78 | 61.11 | 56.25 | 83.33 | 45.83 | 41.67 |
| **ESC**^19^ | 83.33 | 72.22 | 72.91 | 83.33 | 83.33 | 66.67 |
| **GTH**^20^ | 77.78 | 44.44 | 41.67 | 83.33 | 66.67 | 41.67 |
| **JOGNN**^21^ | 77.78 | 66.67 | 41.67 | 72.22 | 45.83 | 58.33 |
| **Korea**^22^ | 66.67 | 55.56 | 52.08 | 72.22 | 66.67 | 66.67 |
| **RCOG**^23^ | 66.67 | 44.44 | 72.22 | 72.22 | 54.17 | 66.67 |
| **SASTH**^24^ | 66.67 | 61.11 | 54.17 | 83.33 | 66.67 | 41.67 |
| **SOGC**^25^ | 83.33 | 61.11 | 72.91 | 83.33 | 54.17 | 33.33 |
